# Effectiveness of Dexamethasone for COVID-19 in Hospitalized Patients With Diabetes: A Retrospective Cohort Study

**DOI:** 10.1210/clinem/dgae734

**Published:** 2024-10-17

**Authors:** Salman Zahoor Bhat, Jiajun Wu, Jamie Perin, Kunbo Wang, Matthew L Robinson, Brian T Garibaldi, Nestoras Mathioudakis

**Affiliations:** Division of Endocrinology, Diabetes and Metabolism, Department of Medicine, Johns Hopkins University School of Medicine, Baltimore, MD 21287, USA; Department of Pediatrics, Biostatistics, Epidemiology and Data Management (BEAD) Core, Johns Hopkins University School of Medicine, Baltimore, MD 21224, USA; Department of International Health, Johns Hopkins Bloomberg School of Public Health, Baltimore, MD 21205, USA; Department of Applied Mathematics and Statistics, Johns Hopkins University, Baltimore, MD 21218, USA; Division of Infectious Diseases, Department of Medicine, Johns Hopkins School of Medicine, Baltimore, MD 21287, USA; Division of Pulmonary and Critical Care Medicine, Department of Medicine, Johns Hopkins School of Medicine, Baltimore, MD 21287, USA; Division of Endocrinology, Diabetes and Metabolism, Department of Medicine, Johns Hopkins University School of Medicine, Baltimore, MD 21287, USA

**Keywords:** SARS-CoV-2, COVID, glucocorticoids, steroids, diabetes, hyperglycemia, diabetic ketoacidosis

## Abstract

**Background:**

Patients with diabetes have higher mortality from COVID-19 compared to the general population. Dexamethasone, a potent glucocorticoid used for moderate to severe COVID-19, can worsen hyperglycemia in patients with diabetes, potentially leading to worse outcomes. The efficacy and safety of use of dexamethasone for COVID-19 in patients with diabetes needs further evaluation.

**Objective:**

The study aimed to assess the efficacy and safety of dexamethasone in patients with diabetes hospitalized for COVID-19 infection.

**Design:**

This retrospective study analyzed data from 5 hospitals in the Johns Hopkins Health System collected between March 3, 2020, and June 25, 2022. Propensity score matching was applied to a cohort of patients with diabetes who received dexamethasone and those who did not (controls), and outcomes were compared using Cox proportional hazards regression models.

**Outcomes:**

The primary outcome was time to death within 28 days. The secondary outcome was time to clinical improvement. Additional outcomes included the incidence of hyperglycemic emergencies and subgroup analysis of primary outcomes by clinical severity.

**Results:**

Out of 10,329 patients admitted for COVID-19, 3679 had diabetes, and 2361 met the inclusion criteria. After propensity score matching, 529 patients were analyzed in each group. Survival rates between the dexamethasone and control groups during the 0- to 6-day and 7- to 28-day periods and time to clinical improvement at 28 days did not differ significantly. There was no difference in the incidence of diabetic ketoacidosis or hyperosmolar hyperglycemic state between the groups.

**Conclusion:**

Dexamethasone treatment did not significantly improve survival or time to clinical improvement in patients with diabetes and COVID-19 infection. Further prospective studies are needed to confirm these findings and determine potential mechanisms.

The COVID-19 pandemic has resulted in more than 7 million deaths worldwide (1). Patients with diabetes face a significantly higher risk of adverse clinical outcomes; they are twice as likely to develop severe respiratory disease and die from COVID-19 compared to patients without diabetes (2-4). The risk is even greater for those with microvascular and macrovascular complications resulting from diabetes (5, 6). Furthermore, the rates and clinical outcomes of diabetic ketoacidosis (DKA) are worse in COVID-19-infected patients (7-10). Hyperglycemia exacerbates these risks by promoting SARS-COV-2 viral proliferation and adversely affecting pulmonary function. This occurs through an enhanced proinflammatory response, reactive oxygen species generation, and pulmonary vascular dysfunction (11-13). Hyperglycemia weakens the antiviral immune response by impairing the recruitment and function of natural killer and CD-4/CD-8 T-cells (14).

Corticosteroids emerged as one of the early therapies demonstrating clinical benefit in COVID-19-infected patients requiring oxygen therapy. The RECOVERY trial, along with other studies and meta-analyses, confirmed the efficacy of glucocorticoids, leading to recommendations for dexamethasone in the inpatient management of COVID-19 infection (15-17). The RECOVERY trial was a large-scale prospective randomized controlled trial studying the use of 6 mg/day of dexamethasone for 10 days for the management of COVID-19 infection. It reported a 17% reduction in 28-day mortality among SARS-COV-2-infected patients requiring oxygen supplementation [22.9% in the dexamethasone group and 25.7% in the control group; 95% confidence interval (CI) .75 to .93; *P* < .001] (15).

Exacerbation of hyperglycemia in patients with diabetes and the development of new-onset hyperglycemia in patients without diabetes is one of the common side effects of dexamethasone and other potent glucocorticoids (18). Mechanisms of glucocorticoid-induced hyperglycemia include negative postreceptor effects on insulin action, resulting in insulin resistance, increased hepatic gluconeogenesis, and decreased tissue glucose uptake by skeletal muscle and adipose tissue by inhibition of glucose transporter type 4 expression (19-22). Glucocorticoids result in increased glucose production from the liver by stimulating rate-limiting enzymes of hepatic gluconeogenesis and increasing concentrations of gluconeogenic substrates (23). Glucocorticoids impact beta cell function directly by affecting the beta cell lifespan and secretory capacity (24-27). Consequently, this insulin resistance and beta cell dysfunction often lead to substantially increased insulin requirements among insulin-treated patients (28).

Uncontrolled hyperglycemia in patients with COVID-19 infection is associated with a higher likelihood of mechanical ventilation and higher in-hospital mortality (29, 30). Thus, the clinical benefits of dexamethasone in treating COVID-19 in patients with diabetes are not clear-cut and may be offset by its adverse effects on glycemic control. Accordingly, we conducted this study to evaluate the efficacy and safety of dexamethasone for treating COVID-19 in patients with diabetes.

## Materials and Methods

### Study Design and Data Sources

This retrospective cohort study used deidentified data from the Johns Hopkins CROWN database, a registry comprised of electronic medical record data and supplemented by other data sources such as physiologic device monitoring systems of patients. Hosted on the Precision Medicine Analytics Platform, Johns Hopkins CROWN integrates data from 5 hospitals within the Johns Hopkins Health System for the study of COVID-19 (31). The database includes records of patients admitted with symptomatic COVID-19 infection confirmed by SARS-COV-2 polymerase chain reaction testing from nasal or oropharyngeal samples and required hospitalization.

The research question, methodology, and data access received approval from the Johns Hopkins University School of Medicine Institutional Review Board, which also waived the requirement for informed consent due to the minimal risk associated with the study design. Administration of dexamethasone followed World Health Organization (WHO) and Food and Drug Administration guidelines, as recommended by the RECOVERY trial findings (15).

### Study Cohort/Inclusion/Exclusion Criteria

Patients admitted to the Johns Hopkins Health System for the treatment of COVID-19 infection during the period from March 4, 2020, to June 25, 2022, were considered for this study. Eligibility was based on a diagnosis of diabetes identified using International Classification of Diseases, Tenth Revision codes (E10, E11, E12, E13) or a hemoglobin A1C level consistent with diabetes (A1C ≥ 6.5%) either during hospitalization or within 90 days prior. The primary exposure of interest was the administration of oral or IV dexamethasone at a dosage of 6 mg/day in patients who required inpatient management of COVID-19 infection, defined by an oxygen saturation ≤94% on room air or the need for supplemental oxygen or mechanical ventilation to maintain an SpO2 > 94% for at least 1 hour. Exclusions were applied to patients discharged or deceased within 24 hours after hospital admission, those treated with dexamethasone dosages other than 6 mg/day, those with incomplete clinical data, and patients admitted before May 27, 2020, when dexamethasone was not yet a primary treatment for COVID-19. The study aimed to compare outcomes between patients who received dexamethasone and those who did not, after applying inclusion and exclusion criteria.

### Statistical Analysis

Descriptive statistics were used to characterize the study population. The Kruskal–Wallis test assessed continuous variables, while the Fisher test evaluated categorical variables. We performed propensity score matching using both time-independent and time-dependent variables to create clinically comparable pairs of patients, 1 receiving dexamethasone and the other not (32). This matching addressed the nonrandomized assignment of dexamethasone and variable timing of its administration (33).

We calculated the propensity scores using a time-dependent Cox regression model, with the time to dexamethasone receipt as the outcome. For matching, we employed a sequential 1:1 greedy algorithm without replacement from hospital day 0, using a threshold of less than 0.2 times the SD of hazard components for the propensity score difference (33).

The first day of dexamethasone administration was defined as the index day. Time-dependent variables were aggregated daily, recording the mean value for each variable per day. The WHO severity score was computed every 6 hours, recording the highest score of the day for each patient. Missing laboratory data were imputed using the last observation carried forward method if the observation was within 3 days prior; other missing values were addressed with multiple imputation using the chained equations method (34).

Cox proportional hazards regression models, incorporating demographic, clinical, and laboratory variables, analyzed the association between dexamethasone use and study outcomes (32). Further details on matching processes and variables are provided in the Supplemental Data (32).

### Outcomes

The primary outcome was the time to death from the first day of dexamethasone treatment. The secondary outcome was time to clinical improvement from the start of treatment, defined as either hospital discharge without deterioration of the WHO severity score or a reduction of at least 2 points in the WHO severity score during hospitalization up to the maximum follow-up period. We censored the primary outcome data on the lesser of the last day of follow-up or 28 days postmatching. Patients discharged alive were censored at 28 days to accommodate for the competing risks of death and discharge.

Further secondary outcomes included the time to death and clinical improvement stratified by initial WHO severity score: mild/moderate (scores 3-4) and severe/critical (scores 5-7). Additionally, we examined the incidence of DKA and hyperglycemic hyperosmolar state (HHS) in the dexamethasone group compared to the control group (32).

## Results

### Patient Characteristics

**Figure 1.**
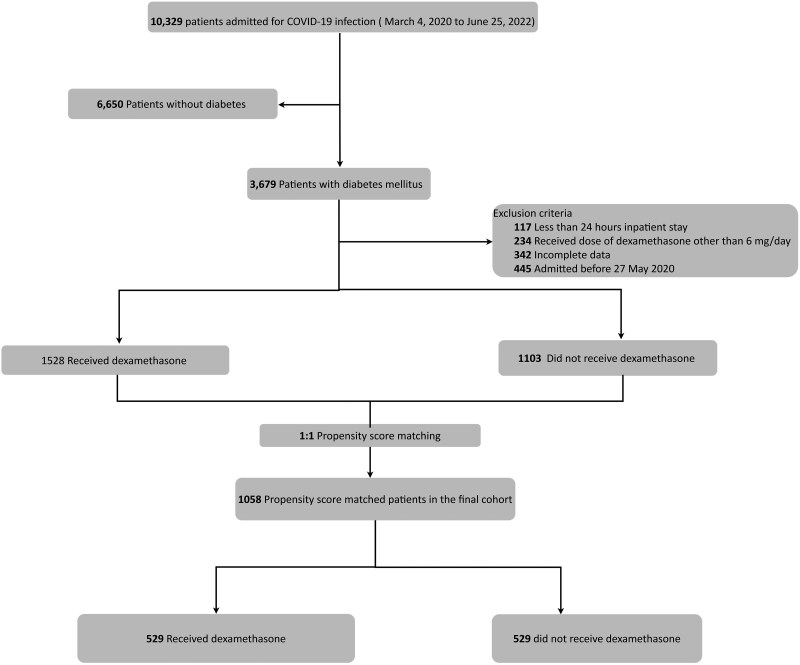
Study flowchart describing patients included in the analysis. *Note: 445 patients who were admitted before May 27, 2020, were removed as dexamethasone was not considered a standard-of-care treatment option for COVID-19 infection prior to this date.

Figure 1 provides the study flowchart. Among 10 329 patients admitted with COVID-19 infection during the study period, we identified 3679 patients with diabetes. After applying the eligibility criteria, the study analyzed data from 2631 patients with diabetes. Among these patients, 1528 received dexamethasone, while 1103 patients did not. The median duration of dexamethasone use was 6 days, the median time from admission to the first dose of dexamethasone was 0.76 days, and median duration between doses was 0.99 days.

The final study cohort for primary analysis was obtained after propensity score matching had 529 patients in the dexamethasone group and 529 patients in the control group. Table 1 shows the baseline characteristics of the study population. Demographics and baseline characteristics were similar between the groups, with a median age of around 65 years and a balanced distribution across sexes and racial groups. The median length of stay and glycemic control parameters (A1C and baseline blood glucose) were also comparable between the 2 groups. The median glucose [interquartile range (IQR)] was 230 mg/dL (168-295) in the treatment group and 185 mg/dL (131, 277) in the control group, with significantly higher levels in the treatment group (*P* < 0.001).

**Table 1.** Baseline characteristics: unmatched and matched cohorts

	All unmatched patients			Propensity score matched patients		
	Dexamethasone	Control	*P*-value	Dexamethasone	Control	*P*-value
Characteristics	n = 1528	n = 1103		n = 529	n = 529	
Sex, number (%)						
Male	810 (53.0)	555 (50.3)		288 (54.4)	273 (51.6)	
Female	718 (47.0)	548 (49.7)	.179	241 (45.6)	256 (48.4)	.388
Ethnicity, number (%)						
Not Hispanic	1354 (88.6)	1004 (91.0)		474 (89.6)	479 (90.5)	
Hispanic	174 (11.4)	99 (9.0)	.052	55 (10.4)	50 (9.5)	.681
Race, number (%)						
Black	608 (39.8)	515 (46.7)		217 (41.0)	222 (42.0)	
White	593 (38.8)	415 (37.6)		202 (38.2)	218 (41.2)	
Other	216 (14.1)	125 (11.3)		69 (13.0)	68 (12.9)	
Asian	111 (7.3)	48 (4.4)	<.001	41 (7.8)	21 (4.0)	.067
Age, y						
Mean (SD)	65.7 (14.8)	63.3 (16.6)	.005	64.5 (16.0)	64.9 (15.3)	.407
Median [IQR]	66.0 [56.0, 76.0]	65.0 [53.0, 75.0]		65.0 [54.0, 76.0]	66.0 [56.0, 77.0]	
Diabetes type						
Type 2	1516 (99.2)	1066 (96.6)	<.001	524 (99.1)	513 (97.0)	.025
Type 1	12 (0.8)	37 (3.4)		5 (0.9)	16 (3.0)	
Code status						
Full code	1429 (93.5)	1042 (94.5)	.323	493 (93.2)	497 (94.0)	.707
DNR/DNI	99 (6.5)	61 (5.5)		36 (6.8)	32 (6.0)	
Length of stay, days	8.00 [5.00, 15.0]	5.00 [3.00, 9.00]	<.001	7.00 [5.00, 11.0]	7.00 [4.00, 12.0]	.337
In-hospital mortality, number (%)	203 (13.3)	43 (3.9)		57 (10.8)	38 (7.2)	.052
Vital signs, median [IQR]						
Temperature, °F	98.6 [97.9, 99.8]	98.2 [97.6, 98.8]		98.5 [97.8, 99.5]	98.2 [97.7, 98.8]	<.001
SpO_2_, %	95.0 [92.0, 97.0]	98.0 [96.0, 99.0]	<.001	95.0 [93.0, 98.0]	97.0 [95.0, 99.0]	<.001
Pulse, beats per min	95.0 [82.0, 108]	91.0 [78.0, 105]	<.001	96.0 [83.0, 110]	91.0 [77.0, 106]	<.001
Respiratory, breaths per min	20.0 [18.0, 24.0]	18.0 [16.0, 20.0]	<.001	20.0 [18.0, 24.0]	18.0 [17.0, 20.0]	<.001
SBP, mm Hg	132 [117, 149]	135 [118, 154]	.007	132 [117, 150]	134 [116, 153]	.629
DBP, mm Hg	74.0 [64.0, 84.0]	75.0 [65.0, 86.0]	.018	75.0 [65.0, 85.0]	74.0 [64.0, 86.0]	.822
BMI, kg/m2	31.0 [27.0, 37.0]	28.0 [24.0, 34.0]	<.001	30.0 [26.0, 36.0]	28.0 [24.0, 35.0]	.031
Glycemic parameters, median [IQR]						
Blood glucose, mg/dL	170 [125, 248]	159 [117, 244]	.011	166 [124, 251]	162 [119, 243]	.157
HbA1C, %	7.50 [6.70, 8.90]	7.60 [6.60, 9.40]	.426	7.30 [6.60, 8.95]	7.50 [6.60, 9.10]	.546
Lab values, median [IQR]						
WBC count, 103/μL	7.06 [5.19, 9.65]	7.82 [5.54, 11.2]	<.001	7.44 [5.50, 10.5]	7.63 [5.41, 11.8]	.24
Platelet count, 103/μL	203 [155, 262]	231 [170, 304]	<.001	216 [158, 283]	222 [162, 293]	.272
D-dimer, μg/mL	1.03 [0.620, 1.91]	1.21 [0.610, 2.42]	.038	1.07 [0.620, 1.98]	1.39 [0.670, 3.12]	.002
Ferritin, μg/L	565 [282, 1060]	388 [140, 845]	<.001	502 [245, 1040]	460 [172, 946]	.087
Comorbidities, number (%)						
Hypertension	1362 (89.1)	973 (88.2)	.492	472 (89.2)	485 (91.7)	.209
Diabetic nephropathy	634 (41.5)	521 (47.2)	.004	209 (39.5)	266 (50.3)	.001
Diabetic retinopathy	254 (16.6)	239 (21.7)	.001	85 (16.1)	114 (21.6)	.027
Coronary artery disease	657 (43.0)	537 (48.7)	.004	228 (43.1)	283 (53.5)	.001
Heart failure	567 (37.1)	440 (39.9)	.155	202 (38.2)	234 (44.2)	.053
Stroke	257 (16.8)	216 (19.6)	.072	93 (17.6)	109 (20.6)	.241
Chronic lung disease	324 (21.2)	201 (18.2)	.06	94 (17.8)	116 (21.9)	.105
Liver disease	265 (17.3)	242 (21.9)	.004	93 (17.6)	122 (23.1)	.032
Malignancy	100 (6.5)	105 (9.5)	.006	31 (5.9)	47 (8.9)	.077
HIV infection	24 (1.6)	28 (2.5)	.089	8 (1.5)	12 (2.3)	.499
Cerebrovascular disease	350 (22.9)	309 (28.0)	.003	125 (23.6)	165 (31.2)	.007
Medication use, number (%)						
Remdesivir	1159 (75.9)	127 (11.5)	<.001	373 (70.5)	82 (15.5)	<.001
Azithromycin	459 (30.0)	106 (9.6)	<.001	143 (27.0)	64 (12.1)	<.001
Tocilizumab	80 (5.2)	3 (0.3)	<.001	11 (2.1)	3 (0.6)	.056
Anticoagulation	1496 (97.9)	977 (88.6)	<.001	515 (97.4)	489 (92.4)	<.001
Hydroxychloroquine	7 (0.5)	2 (0.2)	.319	2 (0.4)	1 (0.2)	1
Statin therapy, outpatient	933 (61.1)	665 (60.3)	.716	292 (55.2)	341 (64.5)	.003
Antihypertensive therapy, outpatient	1130 (74.0)	819 (74.3)	.892	382 (72.2)	403 (76.2)	.16
Insulin therapy, outpatient	634 (41.5)	500 (45.3)	.051	206 (38.9)	254 (48.0)	.004
Diabetic therapy at home						
No antidiabetic therapy recorded	381 (24.9)	279 (25.3)	.001	148 (28.0)	130 (24.6)	.054
Only insulin therapy	234 (15.3)	228 (20.7)		88 (16.6)	115 (21.7)	
Only noninsulin therapy	477 (31.2)	313 (28.4)		164 (31.0)	137 (25.9)	
Combined insulin and noninsulin therapy	400 (26.2)	272 (24.7)		118 (22.3)	139 (26.3)	
Incomplete data	36 (2.4)	11 (1.0)		11 (2.1)	8 (1.5)	
Home GLP-1a use	190 (12.4)	113 (10.2)	.084	61 (11.5)	58 (11.0)	.846
Home SGLT-2i use	173 (11.3)	127 (11.5)	.901	45 (8.5)	63 (11.9)	.084
Home metformin use	687 (45)	448 (40.6)	.028	222 (42)	206 (38.9)	.347
Charlson's Comorbidity Index, number (%)						
0	43 (2.8)	38 (3.4)	.362	10 (1.9)	20 (3.8)	.094
1-4	1116 (73.0)	754 (68.4)	.01	382 (72.2)	342 (64.7)	.01
≥5	369 (24.2)	311 (28.2)	0.021	137 (25.9)	167 (31.6)	0.049

Abbreviations: BMI, body mass index; DBP, diastolic blood pressure; DNR/DNI, do not resuscitate/do not intubate; GLP-1a, glucagon-like peptide 1 analog; HbA1C, hemoglobin A1C; IQR, interquartile range; SBP, systolic blood pressure; SGLT-2i, sodium glucose transport-2 inhibitor; WBC, white blood cell.

## Study Outcomes

### Time to Death

**Figure 2.**
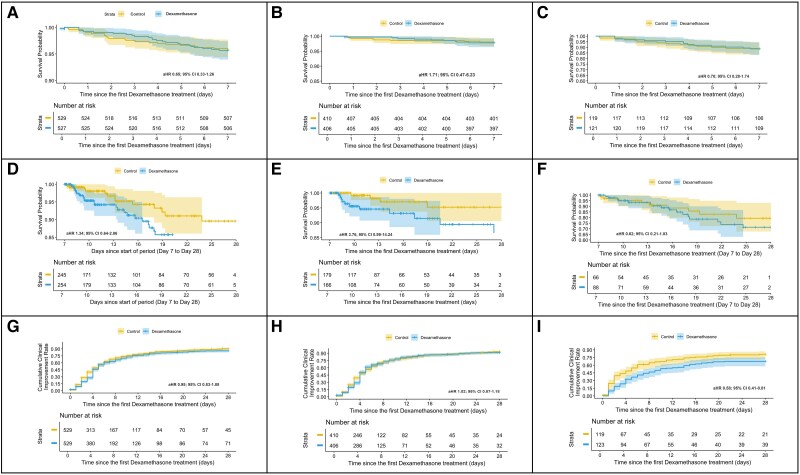
Clinical outcomes. (A-C) Time to death in the 0- to 6-day period in the entire study cohort (A), mild/moderate COVID-19 (B), and severe COVID-19 infection (C), respectively. (D-F) Time to death in the 7- to 28-day period in the entire study cohort (D), mild/moderate COVID-19 (E), and severe COVID-19 infection (F), respectively. (G-I) Cumulative time to clinical improvement in the 0- to 28-day period in the entire study cohort (G), mild/moderate COVID-19 (H), and severe COVID-19 infection (I), respectively. Abbreviation: aHR, adjusted hazard ratio.

The 28-day mortality rates were 8.7% (n = 46) in the dexamethasone group and 6.4% (n = 34) in the control group. The Cox proportional hazard assumption was not maintained across the 28-day period, indicating that the effect of dexamethasone on mortality did not remain constant over time; therefore, in order to adequately assess the temporal effects, the analysis of time-to-death outcomes was segmented into 2 distinct periods: the first covering day 0 to the end of day 6 and the second spanning day 7 to day 28. The segmented analysis of time to death revealed no statistically significant differences between the dexamethasone and control groups during the first period [adjusted hazard ratio (aHR) 0.65; 95% CI .33-1.26, *P* = .20] or the second period (aHR 1.34; 95% CI .64-2.86, *P* = .44) (Fig. 2).

Further subgroup analysis by disease severity showed no significant differences in time to death between the dexamethasone and control groups for patients with mild/moderate COVID-19 infection during both the first period (aHR 1.71; 95% CI .47-6.23, *P* = .418) and the second period (aHR 3.76, 95% CI .99-14.24, *P* = .052) (Fig. 2). Similarly, no significant differences were found for patients with severe/critical COVID-19 infection during the first period (aHR .70; 95% CI .28-1.74, *P* = .44) or the second period (aHR .62; 95% CI .21-1.83, *P* = .39) (Fig. 2).

### Time to Clinical Improvement

Time to clinical improvement was similar in both the dexamethasone group (median 5 days; IQR 2-10 days) and the control group (median 5 days, IQR 3-11 days). At the end of 28 days of follow-up, 84.3% (n = 446) of patients in the dexamethasone group and 89.3% (n = 472) in the control group had achieved clinical improvement. Analysis of time to clinical improvement between 0 and 28 days of follow-up showed no statistically significant benefit in the dexamethasone group compared to the control group (aHR 0.95; 95% CI .83-1.08) (Fig. 2). Subgroup analysis by disease severity revealed no significant reduction in time to clinical improvement with dexamethasone in patients with mild/moderate COVID-19 infection (aHR 1.02; 95% CI .87-1.18); however, in patients with severe/critical COVID-19 infection, a significantly slower time to clinical improvement was observed in the dexamethasone group (aHR 0.58; 95% CI .41-.81) (Fig. 2).

### DKA

The incidence of DKA was not statistically different between the treatment and control groups, with 38 patients (7.2%) in the dexamethasone group and 34 patients (6.4%) in the control group developing DKA (*P* = .714). Among patients who developed DKA, all 38 patients in the treatment group had type 2 diabetes, while in the control group, 5 had type 1 diabetes and 29 had type 2 diabetes.

### HHS

The incidence of HHS was not significantly different between the treatment and control groups, with 26 patients (1.7%) in the treatment group and 19 patients (1.7%) in the control group developing HHS (*P* = 1).

### Sensitivity Analysis

To address potential confounding from the concurrent use of remdesivir, we performed a sensitivity analysis excluding patients from both the treatment and control groups who had received remdesivir. The time-to-death analysis revealed no statistically significant difference during the first period (aHR 1.26; 95% CI .49-3.23, *P* = .63) but indicated a shorter time in the dexamethasone-treated group during the second period (aHR 3.04; 95% CI 1.08-8.57, *P* = .03) compared to the control group, with neither group receiving remdesivir. The time-to-clinical-improvement models did not converge and are therefore not reported. The rates of DKA and HHS were not significantly different between the dexamethasone-only and control groups in the sensitivity analysis [DKA: 7 (4.5%) vs 25 (5.6%), *P* = .683; HHS: 6 (3.8%) vs 8 (1.8%), *P* = .212].

## Discussion

This retrospective cohort study of propensity score-matched patients with diabetes found no statistically significant difference in mortality or clinical improvement between the dexamethasone and control groups. However, among patients with severe/critical COVID-19, dexamethasone use was associated with a slower time to clinical improvement. Additionally, in a sensitivity analysis excluding patients with concurrent use of remdesivir, dexamethasone was associated with a shorter time to death during days 7 to 28 compared to the control group. The incidence of hyperglycemic emergencies, DKA, and HHS, was comparable between the 2 groups.

The COVID-19 pandemic resulted in a surge of hospitalizations and mortality, with dexamethasone emerging as one of the first treatment options to show beneficial clinical outcomes in hospitalized patients. However, COVID-19 infection impacts individuals differently, and patients with diabetes are more likely to experience worse outcomes (3, 35). Regrettably, this cohort also faces a heightened risk of hyperglycemia due to dexamethasone (18, 22).

Our findings offer critical insights for clinicians managing inpatient COVID-19 among patients with diabetes. We observed no overall benefit in reducing mortality or accelerating clinical improvement. Notably, our data indicate that patients with severe/critical COVID-19 who were treated with dexamethasone experienced a significantly slower recovery. The finding of shorter time-to-death with dexamethasone in the sensitivity analysis may reflect an indication bias, where sicker patients were more likely to receive dexamethasone. Given the retrospective nature of our study, we cannot establish causality, and the worse outcomes observed with dexamethasone treatment may be due to residual confounding rather than a direct effect of the medication on recovery rates. These findings underscore the need for further investigation through randomized controlled trials or prospective studies to better understand the implications of dexamethasone treatment in this high-risk patient population.

Our study is among the first to investigate significant clinical outcomes associated with dexamethasone use for COVID-19 infection in patients with diabetes, contributing a unique perspective to the existing literature. A study by Eng et al, which did not utilize propensity score matching, noted similar benefits of dexamethasone use across 2 different waves of COVID-19 infection in patients with and without diabetes (36). Our study benefits from a diverse and large cohort matched on an extensive set of clinical variables impacting outcomes in COVID-19 infection, including often underrepresented populations at high risk for adverse outcomes.

However, several limitations must be acknowledged. The retrospective nature of our study introduces the potential for confounding bias due to the nonadjustment of unknown variables. Furthermore, the final propensity score-matched cohort comprised only 40.3% of the unmatched cohort, introducing a potential for selection bias. This was primarily due to challenges in identifying equally sick patients in the control group, as dexamethasone became a standard of care for severe COVID-19 infection. This limitation may have affected the comparability between the groups, potentially leading to an underestimation of effects due to a reduced statistical power. Additionally, the approval of different therapies at various times during the COVID-19 pandemic introduced significant temporal associations in the use of interventions, complicating the interpretation of outcomes due to these secular trends and the concurrent treatments for COVID-19. A majority (70.5%) of the dexamethasone-treated patients in the final cohort also received remdesivir, whereas only 15.5% of patients who did not receive dexamethasone were treated with remdesivir (15.5%). This disparity in treatment could have influenced the clinical outcomes in the respective groups, although we attempted to address this issue with our sensitivity analysis. The WHO severity scale used to assess COVID-19 severity may not accurately reflect the true range of disease severity, as it tends to group diverse scores under the same severity category. Moreover, it lacks the incorporation of inflammatory markers, which predict severity in newer prediction models, and does not include baseline clinical variables known to affect severity and outcomes (2).

## Conclusions

Our study results suggest that the use of dexamethasone for the management of COVID-19 infection in patients with diabetes may not offer significant benefits in terms of survival or clinical improvement compared to patients who did not receive dexamethasone. Furthermore, an association of worse outcomes was observed in severe COVID-19 patients with diabetes who received dexamethasone. Further randomized controlled trials targeting high-risk patients with diabetes and severe COVID-19 are needed to better understand the risk/benefit ratio of dexamethasone use in this population.

## Data Availability

Restrictions apply to the availability of some or all data generated or analyzed during this study to preserve patient confidentiality or because they were used under license. The corresponding author will on request detail the restrictions and any conditions under which access to some data may be provided.
